# Confined SnO_2_ quantum-dot clusters in graphene sheets as high-performance anodes for lithium-ion batteries

**DOI:** 10.1038/srep25829

**Published:** 2016-05-16

**Authors:** Chengling Zhu, Shenmin Zhu, Kai Zhang, Zeyu Hui, Hui Pan, Zhixin Chen, Yao Li, Di Zhang, Da-Wei Wang

**Affiliations:** 1 State Key Laboratory of Metal Matrix Composites, Shanghai Jiao Tong University, Shanghai 200240, P. R. China; 2 School of Mechanical, Materials & Mechatronics Engineering, University of Wollongong, Wollongong, NSW 2522, Australia; 3 School of Chemical Engineering, UNSW Australia, UNSW Sydney, NSW 2052, Australia

## Abstract

Construction of metal oxide nanoparticles as anodes is of special interest for next-generation lithium-ion batteries. The main challenge lies in their rapid capacity fading caused by the structural degradation and instability of solid-electrolyte interphase (SEI) layer during charge/discharge process. Herein, we address these problems by constructing a novel-structured SnO_2_-based anode. The novel structure consists of mesoporous clusters of SnO_2_ quantum dots (SnO_2_ QDs), which are wrapped with reduced graphene oxide (RGO) sheets. The mesopores inside the clusters provide enough room for the expansion and contraction of SnO_2_ QDs during charge/discharge process while the integral structure of the clusters can be maintained. The wrapping RGO sheets act as electrolyte barrier and conductive reinforcement. When used as an anode, the resultant composite (MQDC-SnO_2_/RGO) shows an extremely high reversible capacity of 924 mAh g^−1^ after 200 cycles at 100 mA g^−1^, superior capacity retention (96%), and outstanding rate performance (505 mAh g^−1^ after 1000 cycles at 1000 mA g^−1^). Importantly, the materials can be easily scaled up under mild conditions. Our findings pave a new way for the development of metal oxide towards enhanced lithium storage performance.

Anode materials with high capacities and excellent cycling stability have been searched for more than ten years^1^,^2^,^3^,^4^,^5^,^6^,^7^,^8^,^9^ to replace the conventionally used graphite which possess a low theoretical capacity of 372 mAh g^−1^ in lithium-ion batteries (LIBs). With high theoretical capacities (~1000 mAh g^−1^), many metal oxides are deemed as the promising materials for next-generation LIB anode^4^,^5^,^6^,^7^,^8^,^9^. Unfortunately, two obstacles exist to hinder their practical applications: one is the intrinsic low electrical conductivity and low Li^+^ diffusion rate in bulk materials, resulting in great mismatch between the actual capacity and the theoretical capacity, especially at high current densities^5^,^10^; the other is the ceaseless generation and rupture of solid–electrolyte interphase (SEI) layer, caused by the pulverization and agglomeration of anode materials due to their significant volume change during lithiation/delithiation process. The proliferous SEI layer gives rise to the continuous consumption of electrolyte and active material, and takes the blame for capacity fading^11^,^12^.

Diminishing the size of anode materials to nanoscale, and further to quantum-dot (QD) scale is proven to be an effective route to shorten the diffusion length of Li^+ ^13. Nevertheless, the specific surface area of particles would rise with the decrease in particle size, causing more exposed surfaces for SEI formation^14^,^15^. Assembling nanoparticles into clusters might be an efficient approach to deal with this problem. In 2014, Cui *et al*. designed and fabricated a porous nanoparticle–cluster structure in Si-based LIB anode^16^. SiO_2_ sacrificial layer was employed to create enough void space around individual Si nanoparticles, which were then encapsulated by amorphous carbon through an emulsion process and the subsequent pyrolysis of resorcinol–formaldehyde resin. The carbon shell provides both an effective barrier against excessive formation of SEI and high electrical conductivity. Unfortunately, the emulsion operation used is complex. The exclusive employment of SiO_2_ sacrificial layer to create void space around individual Si particles and the subsequent unhandy HF etching process, limit the application of this elaborate Si-based anode, and make it a great challenge to apply the synthesis route in other anode materials, such as SnO_2_. Moreover, the insufficient conductivity of the amorphous carbon coating could be improved further.

Herein, we develop an easy and cheap route of fabricating a novel and hierarchical structure of SnO_2_-based anode, in which SnO_2_ QD clusters with internal mesopores are wrapped dexterously with reduced graphene oxide sheets (denoted as MQDC-SnO_2_/RGO). The superiority of this novel structure lies in the multiple virtues: (1) the ultra-small size (~3 nm) of the SnO_2_ QDs and the mesopores assembled in clusters of micrometer size would shorten the diffusion length of Li^+^ and provide extensive surface for lithiation/delithiation reaction; (2) the amorphous carbon matrix around the SnO_2_ QDs and the RGO shell outside the clusters greatly reinforce the electronic conductivity of the composite; (3) the SnO_2_ QDs are intactly encapsuled in the dual carbon coating layers (amorphous carbon and RGO), which restrain the formation of SEI. When used as anode material, the resultant MQDC-SnO_2_/RGO shows superior performances.

## Results

**Figure 1:**
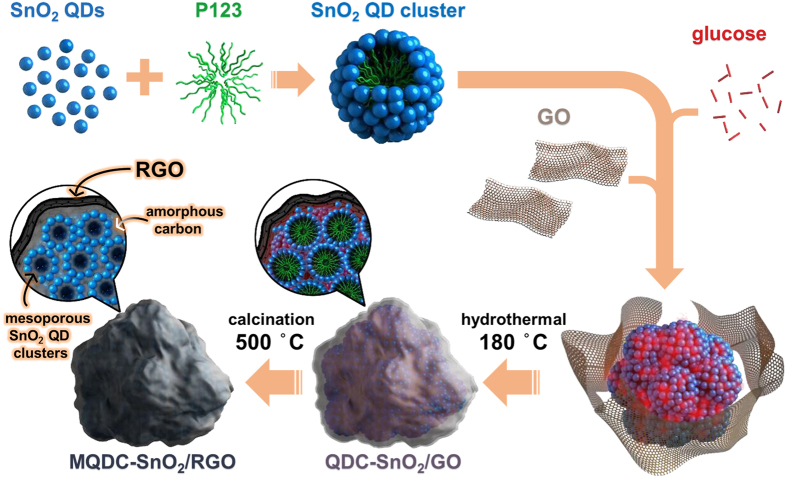
Schematic of the synthesis route of MQDC-SnO_2_/RGO.

The synthesis route of MQDC-SnO_2_/RGO is illustrated in Fig. 1. Firstly, SnO_2_ QDs were prepared in aqueous environment at room temperature (see Supplementary Fig. S1 online), resulting in nanoparticles of ~3 nm as observed by TEM and HRTEM (Supplementary Fig. S2), close to the computed value from the X-ray diffraction (XRD) (Supplementary Fig. S3, Table S1). The strong quantum confinement effect detected by ultraviolet-visible (UV-vis) absorption spectroscopy indicates that the nanoparticles are quantum-dots (Supplementary Fig. S4). Then P123 was added, causing the spontaneous formation of SnO_2_ QD clusters in hydrosol, as detected by dynamic light scattering (DLS) (Supplementary Fig. S5). Subsequent introduction of graphene oxide (GO) and glucose were performed, and hydrothermal process was then conducted to partly reduce GO with glucose as reductant^17^,^18^, so as to induce π–π stacking between RGO sheets and reach a wrapping effect on the surface of SnO_2_ QD clusters. The composite was denoted as QDC-SnO_2_/GO after hydrothermal process. QDC-SnO_2_/GO was calcined under N_2_ to pyrolyze P123 and convert glucose to carbon. The pyrolysis of P123 creates mesopores between SnO_2_ particles and the carbon transformed from glucose encapsulates the SnO_2_ QDs. Thus the resultant MQDC-SnO_2_/RGO composite was obtained.

**Figure 2:**
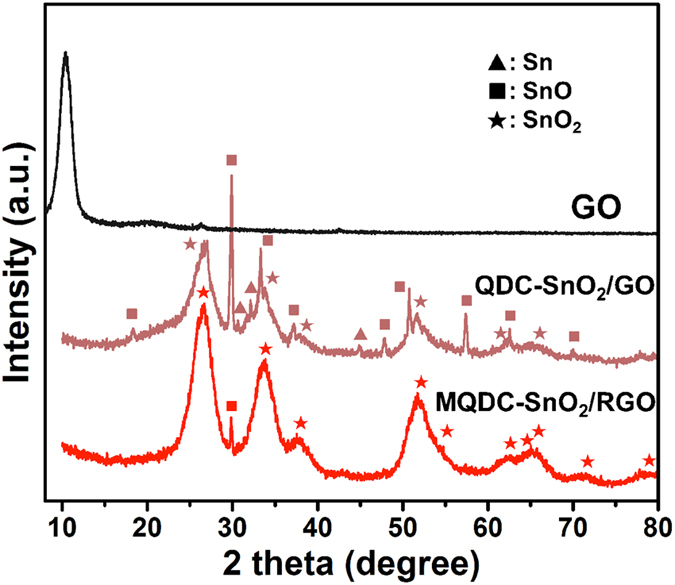
XRD pattern of GO, MQDC-SnO_2_/RGO and QDC-SnO_2_/GO.

The composition of MQDC-SnO_2_/RGO was determined with XRD (Fig. 2). The peaks on the XRD pattern of the composite before calcination, QDC-SnO_2_/GO, can be assigned to SnO_2_, SnO and a small quantity of Sn. The elemental Sn is attributed to the reducing effect of glucose during hydrothermal process. At the same time GO is reduced to RGO partly also, seen from the absence of the characteristic peak of GO at 10.5°. After calcined at 500 °C, it can been found from the XRD pattern of MQDC-SnO_2_/RGO that Sn and most SnO convert to SnO_2_, leaving only negligible quantity of SnO.

**Figure 3:**
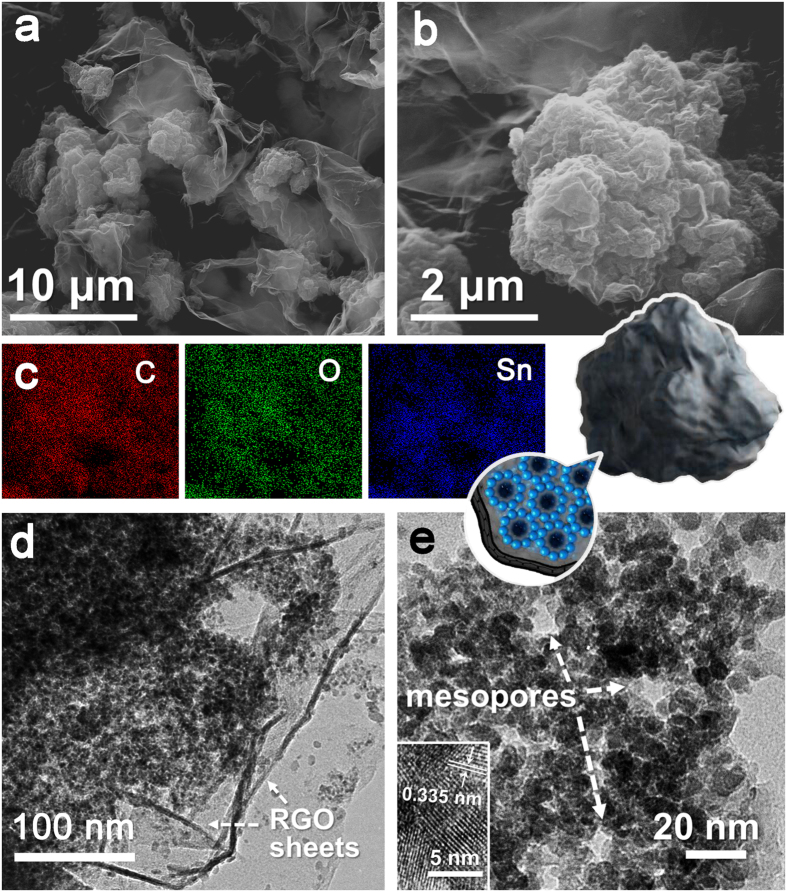
(**a**,**b**) Secondary electron images of MQDC-SnO_2_/RGO in different magnifications. (**c**) Elemental mapping of MQDC-SnO_2_/RGO corresponding to the area in (**a**). (**d**,**e**) TEM images of MQDC-SnO_2_/RGO.

Secondary electron images under scanning electron microscope (SEM) (Fig. 3a,b) reveal the surface morphology of MQDC-SnO_2_/RGO. Clusters of 3–5 μm have been wrapped tightly by RGO sheets, with a plicated appearance. It appears that the distribution of SnO_2_ in RGO sheets is fairly even in the energy-dispersive X-ray elemental mapping result (Fig. 3c). No scattered SnO_2_ particle outside the clusters was found in the secondary electron images. The fine nano-structure of MQDC-SnO_2_/RGO is further investigated by TEM (Fig. 3d,e and Supplementary Fig. S7). The SnO_2_ QD clusters wrapped by thin RGO sheets (with lots of wrinkled area) can be clearly observed (Fig. 3d). Within the clusters there are plentiful pores with pore size under 10 nm as shown in Fig. 3e.

It should be mentioned that glucose plays an indispensable role in the formation of this wrapped cluster structure. As a comparison, a sample prepared under the same condition as MQDC-SnO_2_/RGO but without the addition of glucose is denoted as T-SnO_2_/RGO. Contrast to MQDC-SnO_2_/RGO, T-SnO_2_/RGO demonstrates a flat and dense surface, with SnO_2_ QDs tiled on RGO sheets, while no cluster structure can be found from its SEM images (Supplementary Fig. S6). As glucose molecules are of abundant hydroxyl groups, they tend to form hydrogen bonds with the oxygen atoms on the surface of SnO_2_ QDs and the epoxy groups on GO sheets^17^. During the hydrothermal process, these bonds can crosslink GO sheets and SnO_2_ QDs and stabilize the cluster structure of SnO_2_ QDs. After calcination, glucose decomposes into amorphous carbon that covers on the surface of SnO_2_ QDs, and thus the cluster structure forms in MQDC-SnO_2_/RGO, as shown in Fig. 3a.

**Figure 4:**
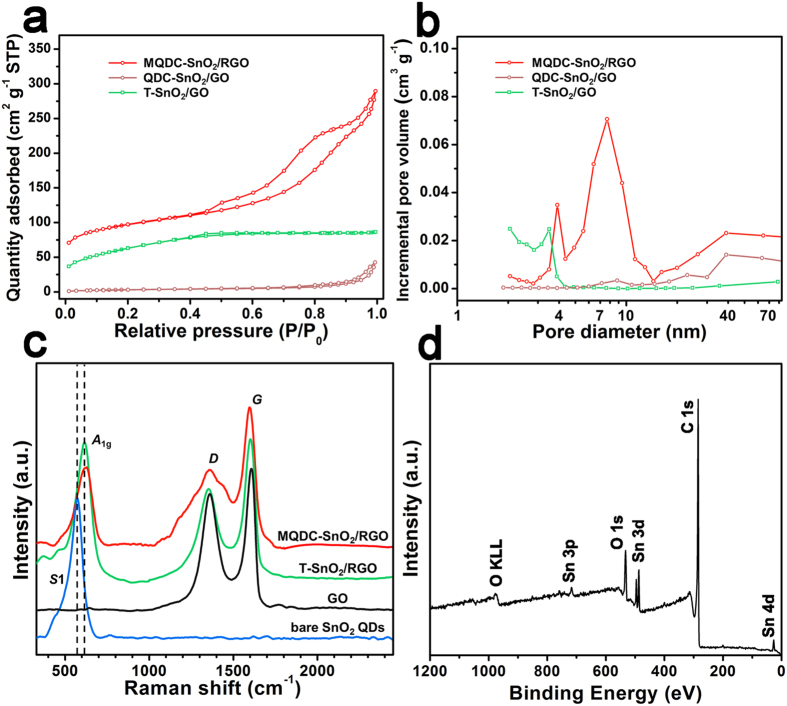
(**a**) Nitrogen adsorption/desorption isotherms and (**b**) pore size distribution of MQDC-SnO_2_/RGO, QDC-SnO_2_/GO and T- SnO_2_/RGO. (**c**) Raman spectra of MQDC-SnO_2_/RGO, T-SnO_2_/RGO, GO and bare SnO_2_ QDs. (**d**) Survey XPS spectra of MQDC-SnO_2_/RGO.

The Brunauer–Emmett–Teller (BET) analysis results of nitrogen adsorption measurements show that MQDC-SnO_2_/RGO has a high specific surface area (SSA) of 337 m^2^ g^−1^ (Fig. 4a). Moreover, the bi-distribution of the mesopores is found in Fig. 4b, one at 4 nm and another at 8 nm. The pores with size of 8 nm should be attributed to the addition of P123^19^. The formation of the smaller-size pores (4 nm) is deemed to be related to the native space among SnO_2_ QDs. It is deduced that P123 decomposes at 500 °C, leaving large amounts of mesopores in MQDC-SnO_2_/RGO. As a comparison, T-SnO_2_/RGO has an SSA of 230 m^2^ g^−1^, while mesopores around 8 nm cannot be found. Because P123 was also added during the synthesis process, the lack of pores around 8 nm in T-SnO_2_/RGO undoubtedly results from the absence of glucose. Without the reinforcement of the amorphous carbon derived from glucose, the pores left by P123 in T-SnO2/RGO collapse completely during the calcination process.

The carbon materials in MQDC-SnO_2_/RGO have been characterized using Raman spectroscopy (Fig. 4c). The intensity ratio of *D* band (~1350 cm^−1^) and *G* band (~1582 cm^−1^) (denoted as *I*_*D*_*/I*_*G*_) has long been introduced as an index of disorder in carbon materials and also a reference to check the extent of the reduction of GO^20^,^21^,^22^. *I*_*D*_*/I*_*G*_ for MQDC-SnO_2_/RGO and T-SnO_2_/RGO were calculated to be 0.58 and 0.65, respectively. They are much smaller than that of the GO counterpart (0.82) and thus indicate that the GO was reduced to RGO in the synthesis. The broadening of *D* band of MQDC-SnO_2_/RGO should be attributed to the glucose-derived amorphous carbon^23^, which is not observed in that of T-SnO_2_/RGO. The *S*1 band (~570 cm^−1^) of bare SnO_2_ QDs shifts to a new position that centers at mode *A*_*1g*_ (~630 cm^−1^) in the spectra of MQDC-SnO_2_/RGO and T-SnO_2_/RGO. This shift is caused by slightly grain growth and the higher crystallinity of SnO_2_ QDs after calcination^24^.

The content of SnO_2_ in MQDC-SnO_2_/RGO was measured to be 48 wt% by thermogravimetric analysis (TGA) (Supplementary Fig. S8). However, it was found in the X-ray photoelectron spectroscopy (XPS) survey spectrum of MQDC-SnO_2_/RGO that the peak intensity of C 1s is much stronger than Sn 3d, 4d and 3p peaks (Fig. 4d), and the mass concentrations of C and Sn elements were computed to be 82.40% and 8.06%, respectively (Supplementary Table S2). This result shows a great disparity with the TGA result. This is unsurprising as the photoelectron signal detected by XPS is generally restricted at the surface of samples. Since the SnO_2_ QDs in MQDC-SnO_2_/RGO are wrapped by amorphous carbon and RGO, the signals from Sn could not be clearly detected by XPS. Therefore, the undervalued content of Sn from the XPS result is deemed as an indirect, yet forceful proof of the unique SnO_2_-wrapped-in-RGO structure.

Clearly, the synthesized MQDC-SnO_2_/RGO has the structure of mesoporous SnO_2_ QD clusters wrapped internally with amorphous carbon and externally with RGO sheets. Although MQDC-SnO_2_/RGO has such a sophisticated structure, its synthesis route is practically simple and without involving any complex process like emulsion operation or using a template.

**Figure 5:**
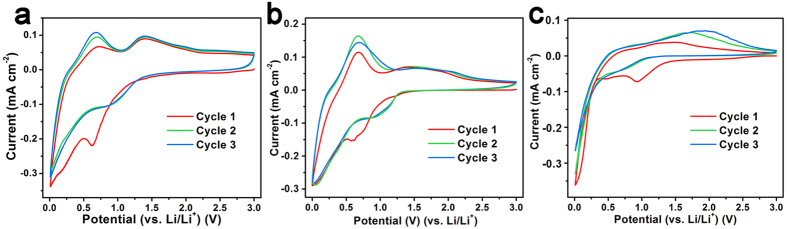
The CVs of (**a**) MQDC-SnO_2_/RGO, (**b**) T-SnO_2_/RGO, and (**c**) bare SnO_2_ QDs, recorded at scan rate of 0.05 mV s^−1^.

The electrochemical performances have been tested by assembling the materials into coin-type half cells. Figure 5 represents the corresponding cyclic voltammograms (CVs). The reduction peaks between 0.5 V to 1.0 V in the first cycle of MQDC-SnO_2_/RGO, T-SnO_2_/RGO and bare SnO_2_ QDs are the sign of the SEI phase formation, ascribed to the reduction of SnO_2_ to Sn by Li^+^, which is usually regarded to be irreversible. However, the stable oxidation peak at 1.40 V and retained reduction peak at 0.95 V in the succeeding cycles of MQDC-SnO_2_/RGO indicate a partial reversibility of the conversion between SnO_2_ and Sn. The partial reversibility can contribute additional capacity to the theoretical base (782 mAh g^−1^)^25^,^26^, but it is not observed in the CV of bare SnO_2_ QDs and is inconspicuous in that of T-SnO_2_/RGO. The theoretically reversible alloying/dealloying reaction of Li_x_Sn is recognized as the reduction peak at 0.01 V and oxidation peak at 0.68 V. This reduction peak gradually weakens for bare SnO_2_ QDs but maintains steady for MQDC-SnO_2_/RGO and T-SnO_2_/RGO. Moreover, the oxidation peak at 0.68 V for MQDC-SnO_2_/RGO shows a gradual upshift compared to that of the bare SnO_2_ QDs. This upshift indicates very different electrochemical properties of MQDC-SnO_2_/RGO from that of bare SnO_2_ QDs.

**Figure 6:**
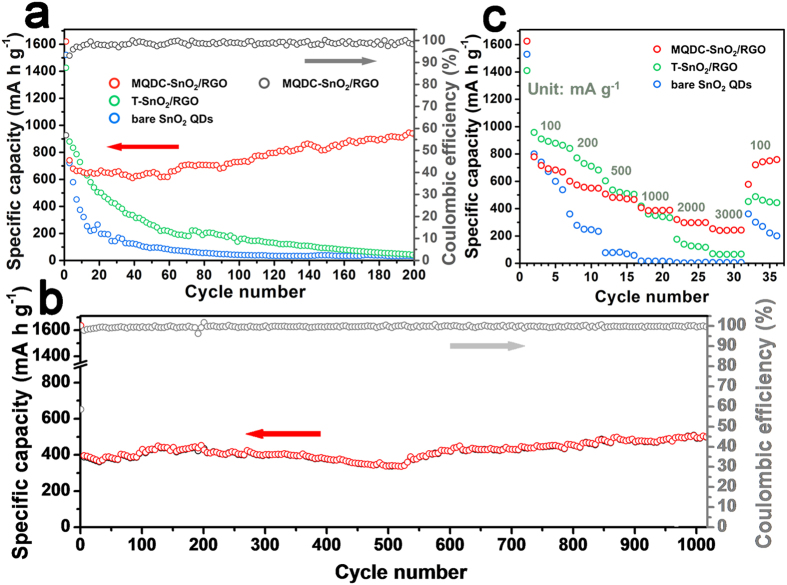
(**a**) Specific discharge capacity and coulombic efficiency of MQDC-SnO_2_/RGO at 100 mA g^−1^, in comparison to the performances of T-SnO_2_/RGO and bare SnO_2_ QDs at the same current density. (**b**) Cyclic performance of MQDC-SnO_2_/RGO at 1000 mA g^−1^ (5 charge/discharge cycles at 100 mA g^−1^ were conducted initially as an activation process). (**c**) Rate performance of MQDC-SnO_2_/RGO, T-SnO_2_/RGO and SnO_2_ QDs (discharge capacities). The varying current is 100 mA g^−1^ for 6 cycles, 200, 500, 1000, 2000 and 3000 mA g^−1^ for 5 cycles each, and finally 100 mA g^−1^ for 5 cycles.

Galvanostatic charge/discharge cycling results are shown in Fig. 6a. For bare SnO_2_ QDs, the initial discharge and charge capacities reached 1522 and 785 mAh g^−1^ respectively, but declined rapidly afterwards and retained only 33 mAh g^−1^ after 200 cycles. Although the ultra-small size of SnO_2_ QDs would drastically shorten the diffusion length of Li^+^ and lead to high specific capacities during the starting several cycles, its quantum confinement effect may cause an augment of charge transfer resistance at the same time^27^. More seriously, the ultra-small size provides large surface area for the formation of SEI, and the bare morphology cannot stand severe pulverization and agglomeration. Similar results were obtained for T-SnO_2_/RGO. The MQDC-SnO_2_/RGO composite with the novel nano-structure is designed to break these limitations. The discharge and charge capacities reach 1620 and 960 mAh g^−1^ in the first cycle, fade to 640 mA g h^−1^ after 10 cycles, and then exhibit a fluctuant recover extending along the subsequent cycles, seen more clearly in the voltage-specific capacity profile (Supplementary Fig. S10). This may be resulted from the novel nano-structure of MQDC-SnO_2_/RGO compared to bare SnO_2_ QDs and T-SnO_2_/RGO. As the SnO_2_ QD clusters are tightly wrapped by RGO sheets and hold a porous internal structure, the compact RGO wrap stabilizes SEI layers and confines them outside the wrapped clusters, and the ample pore space can sustain the significant volume change of SnO_2_ QDs, and thus lead to a stable structure of the SnO_2_ QD clusters. The RGO-wrapped structure of MQDC-SnO_2_/RGO is well retained during its service, attested by the SEM observation after 100 cycles (Supplementary Fig. S11). The charge capacity of MQDC-SnO_2_/RGO reached 924 mAh g^−1^ after 200 cycles (that is 96% of the first cycle capacity, and 144% of the 10^th^ cycle), much higher than the theoretical specific capacity of either SnO_2_ (782 mAh g^−1^) or graphene (744 mAh g^−1^). It is probably due to the partial reversible conversion reaction between SnO_2_ and Sn that verified by the CV results (Fig. 5). Similar fade-recover results were also obtained at a higher charge/discharge current of 1000 mA g^−1^, where a high specific capacity of 505 mAh g^−1^ and a high coulombic efficiency up to 99.8% are retained after 1000 cycles (Fig. 6b). Furthermore, the capacities, the cyclic stability and the rate performance of MQDC-SnO_2_/RGO are dominant to many SnO_2_/graphene anode materials with similar structure to T-SnO_2_/RGO reported in literatures (Supplementary Table S3), and the superiority of MQDC-SnO_2_/RGO as LIB anode is convincingly demonstrated.

The rate performance of MQDC-SnO_2_/RGO is much superior to bare SnO_2_ QDs and T-SnO_2_/RGO, as shown in Fig. 6c. The discharge capacities of the bare SnO_2_ QDs are comparable to those of MQDC-SnO_2_/RGO in the first few cycles, and in the first 16 cycles at 100, 200, and 500 mA g^−1^, the discharge capacities of T-SnO_2_/RGO are even higher than MQDC-SnO_2_/RGO. However, the discharge capacities of bare SnO_2_ QDs and T-SnO_2_/RGO fade rapidly to only 6 and 65 mAh g^−1^ respectively at 3000 mA g^−1^ after 28 cycles while MQDC-SnO_2_/RGO reaches 240 mAh g^−1^ at the same current. Though the capacity of SnO_2_ QDs recovers to 362 mAh g^−1^ when the current is brought down back to 100 mA g^−1^, it fades badly to 201 mAh g^−1^ in 5 cycles, and a similar fading process was also obtained for T-SnO_2_/RGO. However, the ingenious structure of MQDC-SnO_2_/RGO induces a stable capacity recovery (759 mAh g^−1^) when the current is brought down, which is distinct from the bare SnO_2_ QDs or T-SnO_2_/RGO. It is inferred that during the cycles at large currents, the mesopores in SnO_2_ QD clusters provide fast access to Li^+^ while the well conductive RGO sheets give fast access to electrons. The synergistic effect promotes sufficient lithiation/delithiation reaction and maintains the structural integrity of the composite. This echoes the rate performance of the graphene-based mesoporous SnO_2_ composite previously reported^28^. However, the capacity recover phenomenon exhibited by MQDC-SnO_2_/RGO and profitted from its unique structure has not been reported by other researchers.

**Figure 7:**
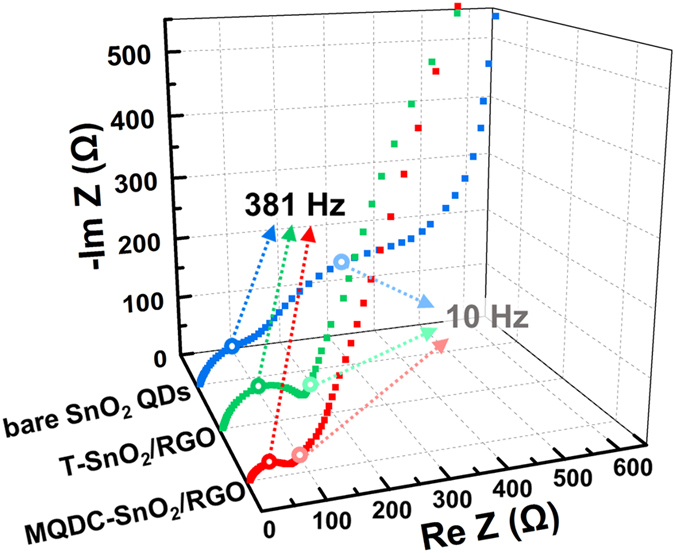
Nyquist plots of MQDC-SnO_2_/RGO, T-SnO_2_/RGO and bare SnO_2_ QDs, measured by EIS with an amplitude of 5.0 mV and the frequency ranging from 100 kHz to 0.01 Hz, after 3 cycles of galvanostatic charge/discharge at 100 mA g^−1^.

Electrochemical impedance spectra (EIS) of MQDC-SnO_2_/RGO, T-SnO_2_/RGO and bare SnO_2_ QDs give corroborative evidences of the electrical conductivity provided by RGO sheets, and the suppressed stable formation of SEI in MQDC-SnO_2_/RGO after charge/discharge cycles. As shown in the Nyquist plots (Fig. 7), the semicircle in the high-medium-frequency region of bare SnO_2_ QDs obviously separates into two semicircles, one in the high-frequency region and another in the medium-frequency region. The medium-frequency semicircle is related to the charge-transfer resistance inside an electrode^29^, which can be obviously observed in the Nyquist plot of bare SnO_2_ but not observed in that of MQDC-SnO_2_/RGO or T-SnO_2_/RGO. This trait indicates a dominant electrical conductivity of the RGO-composed samples to bare SnO_2_ QDs. MQDC-SnO_2_/RGO and T-SnO_2_/RGO each has one semicircle in the high-frequency region and the former has the smallest one. The high-frequency region is related to the diffusion of Li^+^ in SEI layer, and the smaller this semicircle is, the less SEI is in the system^9^. Hence, the formation of the SEI layer was significantly suppressed in MQDC-SnO_2_/RGO because it has the smallest semicircle in the high frequency region among all the three samples. The combination of less SEI layer and higher electrical conductivity resulted in the excellent electrochemical performances of MQDC-SnO_2_/RGO.

## Discussion

In conclusion, an ingenious RGO-wrapped mesoporous SnO_2_ QD cluster composite (MQDC-SnO_2_/RGO) for LIB anode has been designed and successfully fabricated. In this novel architecture, the ultra-small size of SnO_2_ QDs down to ~3 nm surrounded with the mesopores in the SnO_2_ QD clusters can provide short diffusion length of Li^+^ and the RGO sheets give fast access to electrons and depress the formation of SEI, leading to a stable structure. These two factors let MQDC-SnO_2_/RGO have a much high reversible capacity of 924 mAh g^−1^ after 200 cycles at 100 mA g^−1^, and excellent rate performance, reaching 240 mAh g^−1^ at 3000 mA g^−1^. Importantly, the synthesis of the material can be easily scaled up at room temperature, which is a milestone for its practical use. This work opens a new route for the design and construction of other metal oxide clusters with enhanced battery performance, such as Fe_3_O_4_, Fe_2_O_3_ and MnO_2_. It should be mentioning that the rate performance of MQDC-SnO_2_/RGO has much room to be improved, which is deemed to be reached by constructing mesopores not only inside the clusters, but also on the RGO shell.

## Methods

### Synthesis of SnO_2_ quantum dots (SnO_2_ QDs)

The SnO_2_ QDs were synthesized as hydrosol by peptizing the precipitate product of reacting tin tetrachloride (SnCl_4_) aqueous solution with ammonium hydroxide (NH_4_OH). In a typical synthesis, SnCl_4_·5H_2_O (17.53 g) was dissolved in deionized water (100 mL), followed by adding ammonium hydroxide (25 wt%, 10 mL) under rapid stirring. The precipitate was separated out, and washed with deionized water several times until its pH = 9.0. Deionized water (20 mL) was added into the washed precipitate, and the white suspension obtained was stirred continuously to accelerate peptization. When the suspension turned semitransparent, the unpeptized part was removed by high-speed centrifugation, and thus the transparent clear SnO_2_ QD hydrosol was obtained.

### Synthesis of MQDC-SnO_2_/RGO composite

GO was prepared by the well-known Hummers method^30^ and dispersed in deionized water (5 g L^−1^). P123 (0.2 g) was dissolved into the SnO_2_ QD hydrosol (100 g L^−1^, 3 mL), and after 10 min of stirring, glucose (0.18 g) and the as-prepared GO suspension (2 mL) was added. The mixture was transferred into a polytetrafluoroethylene autoclave and kept 180 °C for 3 h. Then, the precipitate was lyophilized and calcined in nitrogen at 500 °C to get MQDC-SnO_2_/RGO composite. For the synthesis of tiled SnO_2_ on reduced graphene oxide (T-SnO_2_/RGO), same operations were conducted except the absence of glucose.

### Materials Characterization

XRD patterns were recorded on a Rigaku D/max2550VL/PC system, using Cu *K*α radiation. DLS measurements were operated on Malvern Zetasizer Nano ZS. SEM (JEOL JSM-6360LV, 15 kV), TEM and HRTEM (JEOL 2010, 200 kV) were used to observe the morphology of the samples. Nitrogen adsorption measurements were performed at 77 K on a Micromeritics ASAP 2020. UV-vis absorption spectra were measured on a Perkin-Elmer 330 spectrophotometer. Raman spectra were collected on a Renishaw in Via Raman microscope. TGA was conducted on a Perkin-Elmer TGA-7 Thermal Analyzer under O_2_ flow, with a heating rate of 20 °C min^−1^. XPS spactra were recorded on a Perkin-Elmer PHI-5400 spectrometer, using Mg *K*α radiation as the excitation source.

### Electrochemical Measurements

The samples were assembled into 2016 coin-type half cells with Li^0^ foil as counter electrodes. To prepare working electrode, the samples were mixed with acetylene black and PVDF by a mass ratio of 8:1:1, and coated on a copper foil. Polypropylene membrane (Celgard 2500) was employed as separator. LiPF_6_ (1 M) in ethylene carbonate (EC) and diethyl carbonate (DEC) (1:1, v/v) was used as electrolyte. Cyclic voltammetry and electrochemical impedance spectroscopy tests were done on a CHI-660D electrochemical workstation. Galvanostatic cycling tests were conducted on a LAND-CT2001A battery test system, with a voltage window of 0.01–3.00 V vs. Li/Li^+^.

## Additional Information

**How to cite this article**: Zhu, C. *et al*. Confined SnO_2_ quantum-dot clusters in graphene sheets as high-performance anodes for lithium-ion batteries. *Sci. Rep.* **6**, 25829; doi: 10.1038/srep25829 (2016).

## Supplementary Material

Supplementary Information
