# Supplementary material for: Confined SnO2 quantum-dot clusters in graphene sheets as high-performance anodes for lithium-ion batteries
Source: Sci Rep. 2016 May 16;6:25829. doi: 10.1038/srep25829 (PMC4867637; doi:10.1038/srep25829)
Supplement: Supplementary Information [file srep25829-s1.pdf]

Supplementary Information

Confined SnO_2 quantum-dot clusters in graphene sheets as high-performance anodes for lithium-ion batteries

Chengling Zhu[1], Shenmin Zhu[1,*], Kai Zhang[1], Zeyu Hui[1], Hui Pan[1], Zhixin Chen[2], Yao Li[1], Di Zhang[1], and Da-Wei Wang[3]

[1]State Key Laboratory of Metal Matrix Composites, Shanghai Jiao Tong University, Shanghai, 200240, P. R. China
[2]School of Mechanical, Materials & Mechatronics Engineering, University of Wollongong, Wollongong, NSW 2522, Australia
[3]School of Chemical Engineering, UNSW Australia, UNSW Sydney, NSW 2052, Australia
*smzhu@sjtu.edu.cn

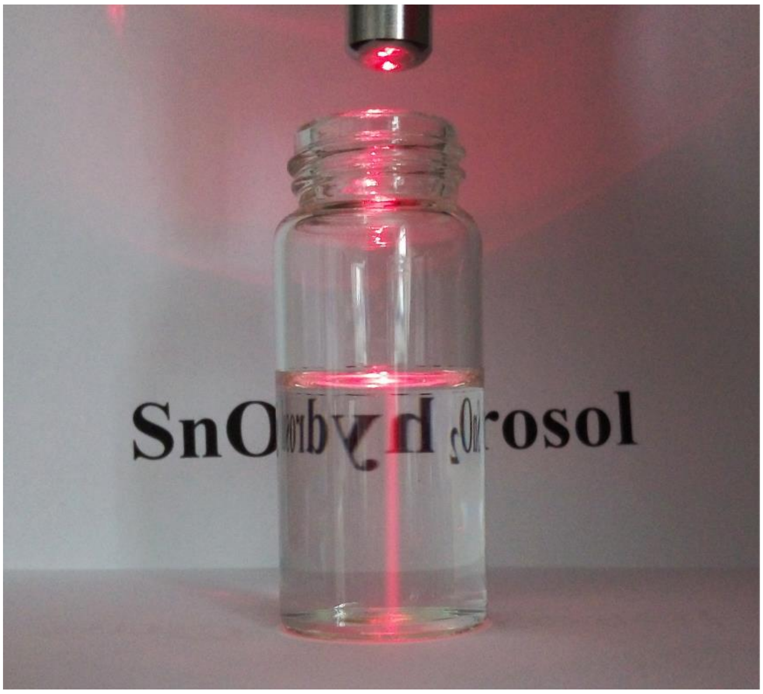

Figure S1. The optical photograph of the as-prepared SnO_2 hydrosol, with a red laser beam shining though it from the top. A clear transparent macroscopic appearance and an obvious Tyndall scattering are observed.

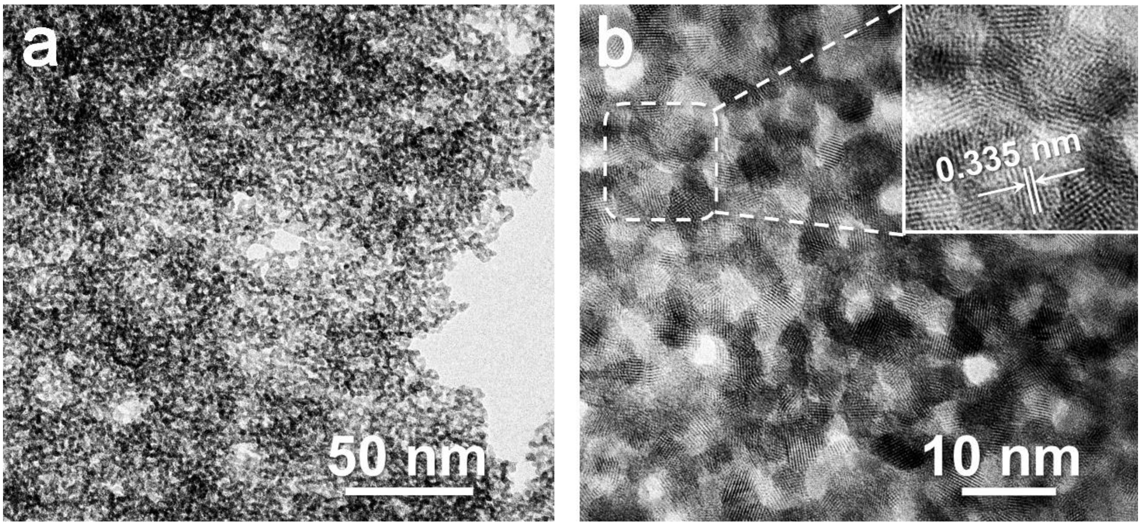

Figure S2. (a) TEM and (b) HRTEM image of the SnO_2 nanoparticles. The SnO_2 nanoparticles are of homogeneous size about 3 nm, close to the computed value from the XRD. The *d*-spacings of 0.335 nm corresponds to the lattice planes (110) of tetragonal SnO_2.

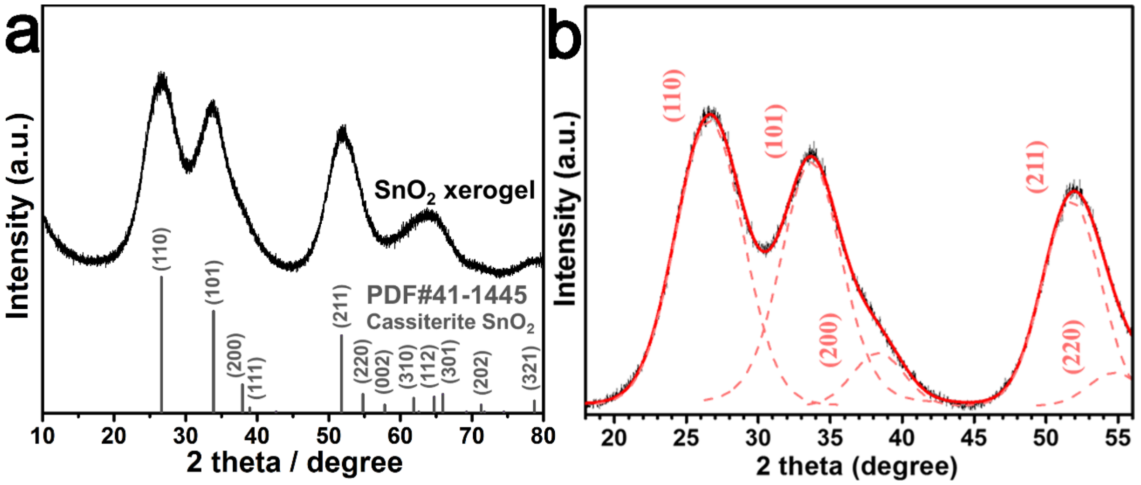

Figure S3. (a) XRD pattern of the SnO_2 xerogel. The diffraction peaks in JCPDS card 41-1445 is marked as grey vertical lines. (b) The peak separation digram of XRD pattern of the SnO_2 xerogel. The original diffraction pattern, the separated fitting diffraction peaks and the summed fitting pattern are plotted in black, red dashed and red solid lines respectively.

Table S1. Mean size of the as-prepared SnO_2 nanoparticles computed from the peak separation fitting result showed in **Figure S1**, using Scherrer equation[1]:

$$\tau = \frac{K\lambda}{\beta\cos\theta}$$

where τ, β, θ respectively represent the mean size of nanoparticles (nm), the line broadening at half the maximum intensity (FWHM) of each peak (rad.) and the Bragg angle, while K is a dimensionless shape factor assigned 0.89 for spherical particles, and λ is the X-ray wavelength utilized (0.154056 nm).

Peak	Position (2θ) (deg.)	FWHM (deg.)	Size (nm)
(110)	26.61	5.60	1.44
(101)	33.89	5.16	1.59
(200)	37.95	4.54	1.83
(211)	51.78	4.78	1.83
(220)	54.76	4.34	2.04
		**average:**	1.75

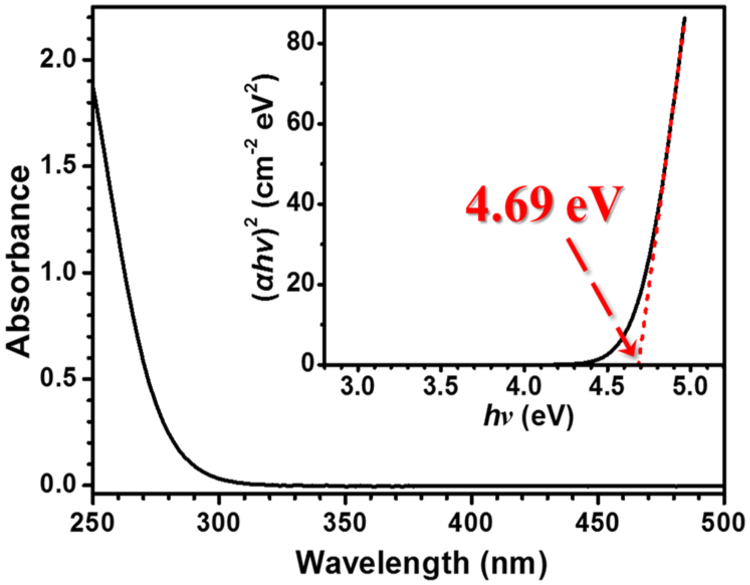

Figure S4. UV-vis absorption spectrum of the SnO_2 hydrosol and the Tauc plot extrapolation digram (inset). As for a semiconductor, the relation between absorption coefficient (α) and light frequency (v) at the edge follows the formula:

$$\alpha hv = A(hv - E_g)^n$$

where h, A and E_g are the Planck constant (6.626×10^{-34} J s), a constant and the optical energy bandgap (eV) respectively, while the value of the exponent n denotes the nature of the transition and herein for direct allowed transitions n = 0.5. Thus by plotting $(\alpha hv)^2$ versus photon energy (hv), E_g can be determined as the intercept on horizontal axis[2], which is 4.69 eV, much larger than the standard bandgap of bulk SnO_2 (3.597 eV)[3]. This huge energy bandgap increase is due to the well-known quantum confinement effect induced from the ultra-small size of the as-prepared SnO_2 nanoparticles[4]. Therefore, the SnO_2 nanoparticles are QDs. In the testing process, the SnO_2 hydrosol was diluted to 1 mg mL^{-1}, and put into a quartz cuvette to get a thickness of 10 mm.

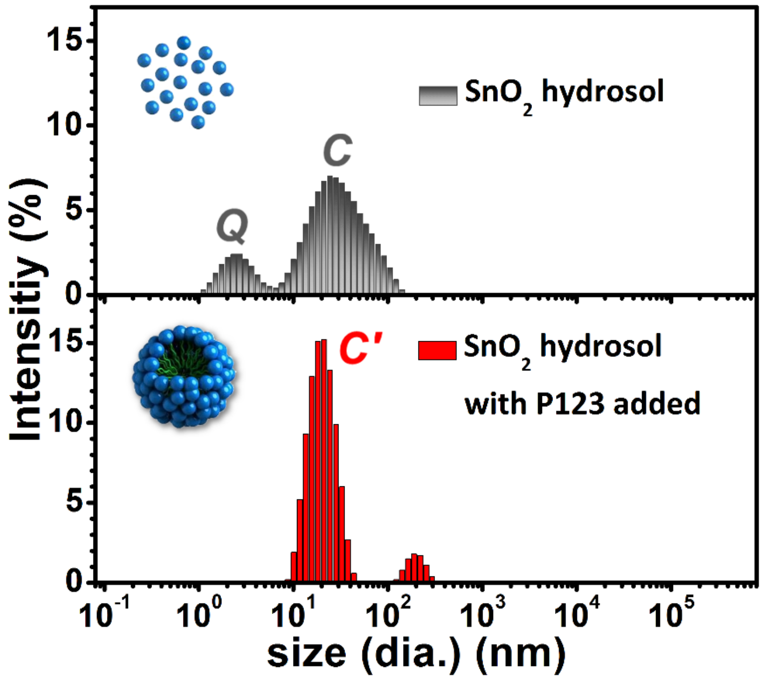

Figure S5. Statistical size distribution histograms of the SnO_2 QDs in 100 g L^{-1} hydrosol without and with P123 added, measured by DLS. The SnO_2 QDs show a dual distribution (two peak areas marked as *Q* and *C*) before the addition of P123. *Q* is centered at 2−3 nm, which agrees well with the monodisperse particle size observed by the XRD and HRTEM results, while the other distribution area *C* (centered at 20−30 nm) is prudently speculated as the formation of SnO_2 QD clusters, as no particle with size over 10 nm is observed under TEM. This phenomenon has been studied previously by Lin *et al.* that monodisperse particles will universally collide and aggregate into clusters[5]. After the addition of P123 into the hydrosol, the size distribution of SnO_2 QDs changes distinctively. The distribution area *Q* disappears, while *C* turns to a narrower distribution peak *C′* (centered at 20 nm). Because intrinsic P123 spherical micelles possess a smaller size of 11 nm[6], *C′* can be interpreted as the formation of a new kind of clusters containing both SnO_2 QDs and P123. This function of P123 that regulate SnO_2 QDs into clusters paves the way for the next step of synthesizing MQDC-SnO_2/RGO.

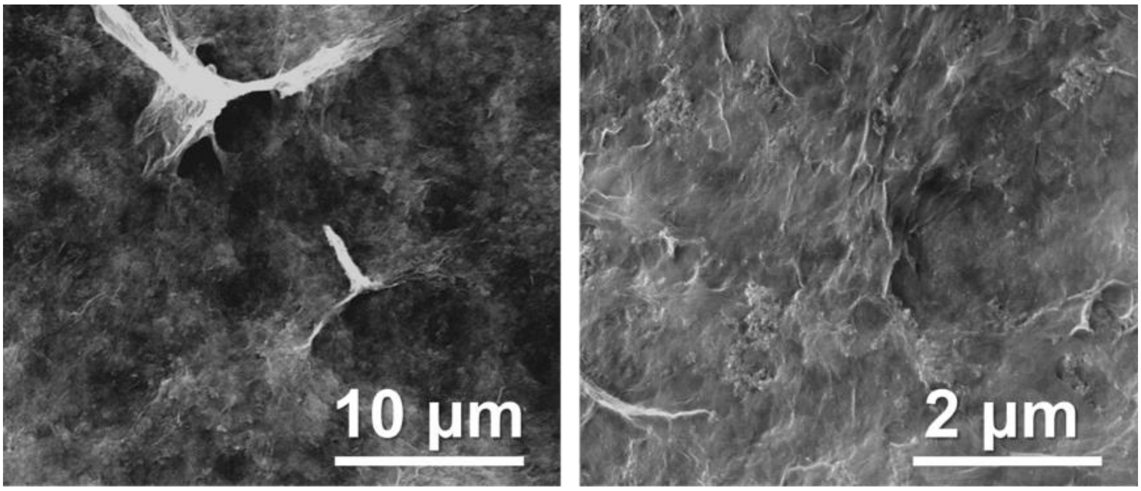

Figure S6. SEM images of T-SnO_2/RGO in different magnifications.

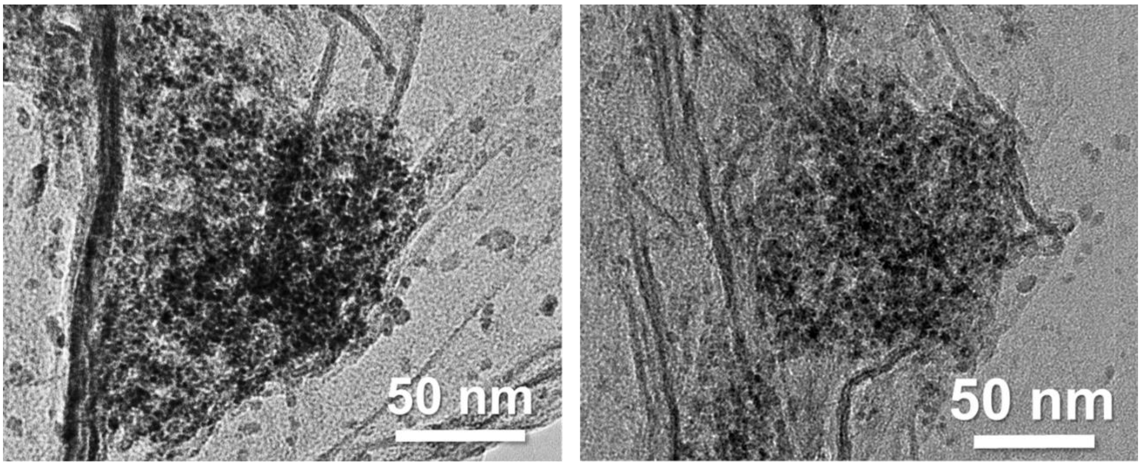

Figure S7. More TEM images of MQDC-SnO_2/RGO. SnO_2 QD clusters wrapped by RGO sheets are widely observed.

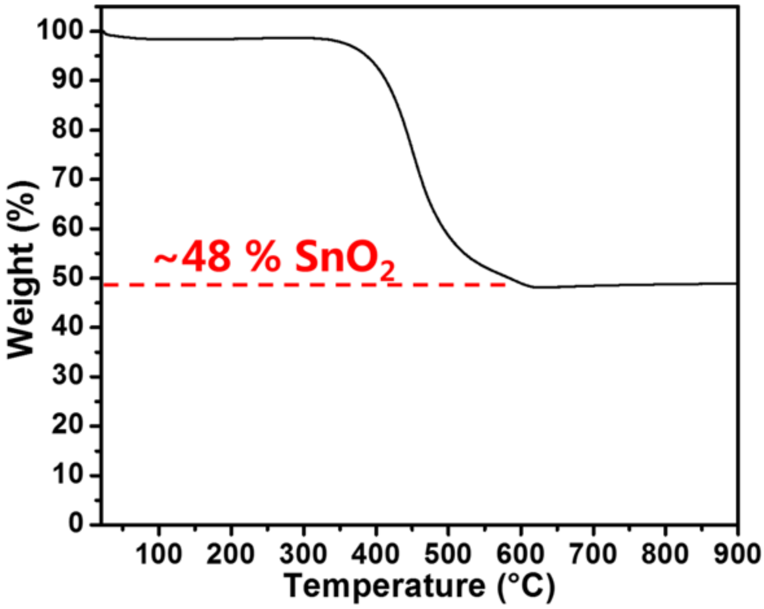

Figure S8. The TGA curve of MQDC-SnO_2/RGO. As the chemical composition of MQDC-SnO_2/RGO is all carbonous materials (RGO and amorphous carbon), SnO_2 and negligible amount of SnO, the residue at 900 ℃ can be regarded as SnO_2 that MQDC-SnO_2/RGO contains, that is ~48 wt%.

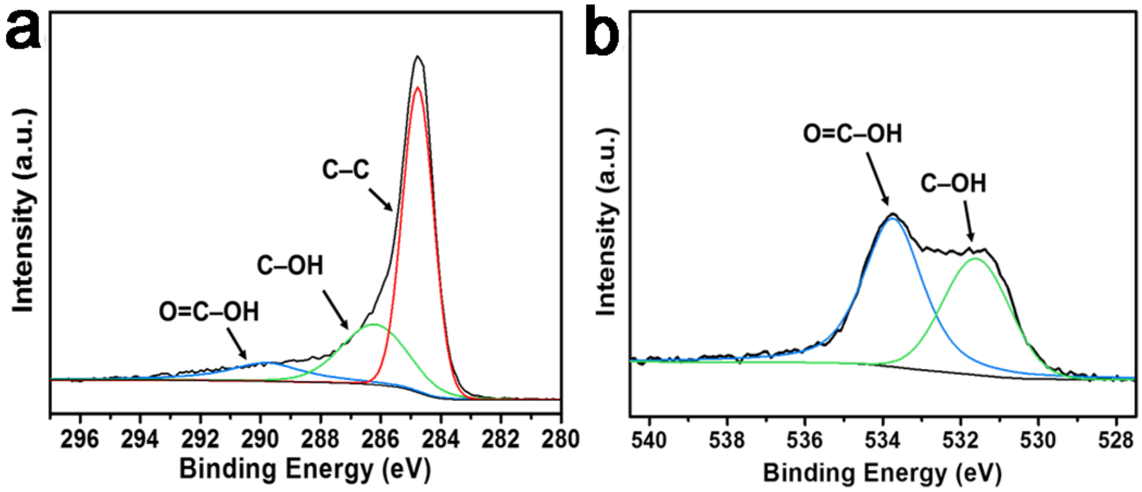

Figure S9. (a) The fine C 1s, and (b) O 1s XPS spectra of MQDC-SnO_2/RGO. After Shirley background subtraction and peak fitting, the spectra give a further confirmation of the reduction of GO sheets. The main C 1s peak located at 284.8 eV is assigned to C–C, the sp^2 C of RGO sheets. The two minor C 1s peaks located at 286.2 and 289.8 eV are assigned to the remnant C–OH and O=C–OH groups formed after calcination, which is in concert with the O 1s peaks located at 531.6 and 533.7 eV.

Table S2. Element contents of C, O, and Sn computed from survey XPS spectra of MQDC-SnO_2/RGO.

Peaks	Position (eV)	FWHM (eV)	Area (T MFP)	Atom Concentration (%)	Mass Concentration (%)
C 1s	284.78	1.278	4852.55	91.17	82.40
O 1s	533.68	3.947	1235.78	7.92	9.54
Sn 3d	487.28	1.355	1204.55	0.90	8.06

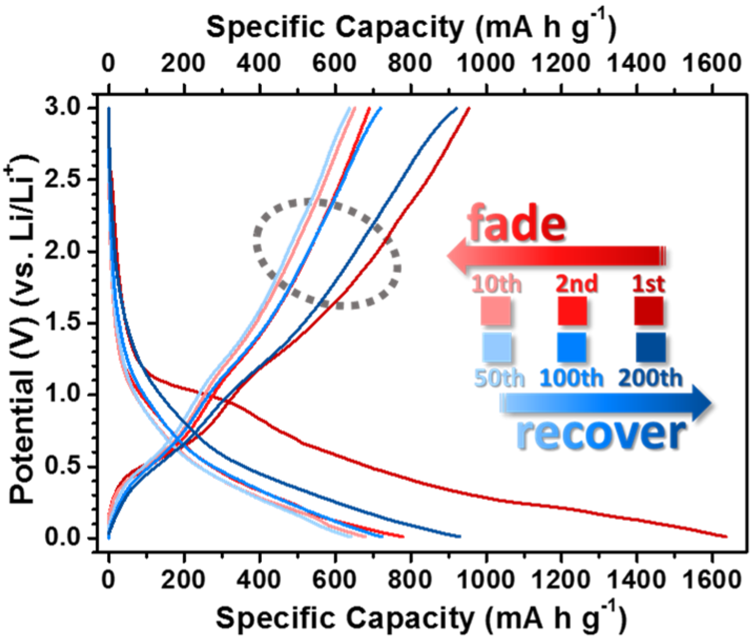

Figure S10. Voltage-specific capacity profile of the 1st, 2nd, 10th, 50th, 100th and 200th galvanostatic charge/discharge cycles of MQDC-SnO_2/RGO at 100 mA g^{-1}.

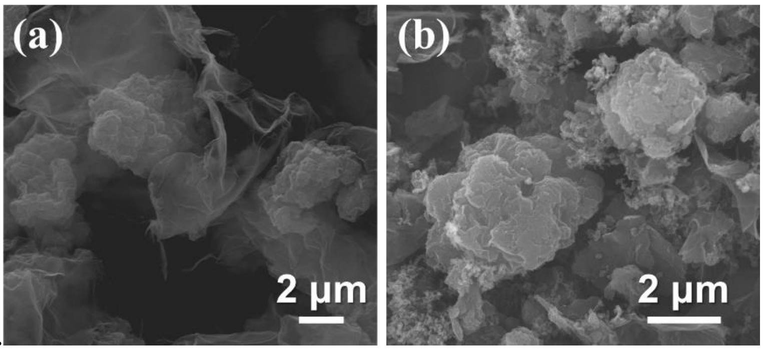

Figure S11. (a) The SEM image of MQDC-SnO_2/RGO before cycling. (b) The SEM image of MQDC-SnO_2/RGO after 100 cycles of galvanostatic charge/discharge at 100 mA g^{-1}.

Table S3. Compare of the capacities, cyclic stability and rate performance of MQDC-SnO_2/RGO with other SnO_2 or SnO_2/graphene LIB anodes reported in literatures.

SnO_2 or SnO_2/graphene LIB anode materials	low rate		high rate	
	Capacity (mA h g^{-1}) / after xx cycles	Current density (mA g^{-1})	Capacity (mA h g^{-1}) / after xx cycles	Current density (mA g^{-1})
**SnO_2 hollow nanostructures[7]**	500 / 40	158	-- / --	--
**SnO_2/graphene nanoporous composite[8]**	570 / 30	50	-- / --	--
**SnO_2-nanocrystal/graphene-nanosheets[9]**	377 / 35	200	-- / --	--
**echinoid-like SnO_2 nanoparticles decorated on graphene[10]**	634 / 50	100	-- / --	--
**reduced graphene oxide/SnO_2 composite[11]**	649 / 30	50	-- / --	--
**graphene nanosheet/SnO_2 composite[12]**	775 / 50	100	-- / --	--
**N-doped graphene-SnO_2 sandwich papers[13]**	910 / 50	50	-- / --	--
**porous SnO_2/graphene composite thin films[14]**	551 / 100	200	-- / --	--
**SnO_2 particles grown on graphene with mesopores[15]**	848 / 50	78	-- / --	--
**SnO_2 nanocrystals bound in graphene oxide[16]**	720 / 200	200	400 / 100	2000
**SnO_2 nanocrystal/graphene composites[17]**	891 / 50	100	-- / --	--
**flower-like SnO_2 nanocrystals distributed on graphene nanosheet[18]**	691 / 40	100	-- / --	--
**ordered network of interconnected SnO_2 nanoparticles[19]**	700 / 35	78	564 / 100	391
**graphene supported SnO_2 particles[20]**	-- / --	--	430 / 140	500
**SnO_2 nanoparticles coated by polyaniline on graphene[21]**	770 / 100	100	350 / 700	1000
**3D macroporous aerogels decorated with SnO_2 particles[22]**	611 / 50	50	-- / --	--
**SnO_2 nanoparticles dispersed on or encapsulated in reduced graphene oxide hybrids[23]**	700 / 100	100	-- / --	--
**3D graphene/carbon nanotube/SnO_2 hybrid[24]**	842 / 40	200	-- / --	--
**ultrathin SnO_2 nanosheets[25]**	758 / 40	200	572 / 40	300
**3D SnO_2/graphene aerogels[26]**	760 / 50	50	-- / --	--
**SnO_2-reduced graphene oxide composites[27]**	770 / 70	100	531 / 1000	1000
**MQDC-SnO_2/RGO in this work**	924 / 200	100	505 / 1000	1000

References:

1. Patterson, A. The Scherrer formula for X-ray particle size determination. *Phys. Rev.* **56**, 978 (1939).
2. Tauc, J. Optical properties and electronic structure of amorphous Ge and Si. *Mater. Res. Bull.* **3**, 37−46 (1968).
3. Nagasawa, M. & Shionoya, S. Exciton structure in optical absorption of SnO_2 crystals. *Phys. Lett.* **22**, 409−410 (1966).
4. Xu, X., Zhuang, J. & Wang, X. SnO_2 quantum dots and quantum wires: controllable synthesis, self-assembled 2D architectures, and gas-sensing properties. *J. Am. Chem. Soc.* **130**, 12527−12535 (2008).
5. Lin, M. *et al.* Universality of fractal aggregates as probed by light scattering. *Proc. R. Soc. London, Ser. A* **423**, 71−87 (1989).
6. Soni, S., Brotons, G., Bellour, M., Narayanan, T. & Gibaud, A. Quantitative SAXS analysis of the P123/water/ethanol ternary phase diagram. *J. Phys. Chem. B* **110**, 15157−15165 (2006).
7. Lou, X. W., Wang, Y., Yuan, C., Lee, J. Y. & Archer, L. A. Template-free synthesis of SnO_2 hollow nanostructures with high lithium storage capacity. *Adv. Mater.* **18**, 2325−2329 (2006).
8. Paek, S.-M., Yoo, E. & Honma, I. Enhanced cyclic performance and lithium storage capacity of SnO_2/graphene nanoporous electrodes with three-dimensionally delaminated flexible structure. *Nano Lett.* **9**, 72−75 (2008).
9. Li, Y., Lv, X., Lu, J. & Li, J. Preparation of SnO_2-nanocrystal/graphene-nanosheets composites and their lithium storage ability. *J. Phys. Chem. C* **114**, 21770−21774 (2010).
10. Kim, H. *et al.* SnO_2/graphene composite with high lithium storage capability for lithium rechargeable batteries. *Nano Res.* **3**, 813−821 (2010).
11. Zhu, X., Zhu, Y., Murali, S., Stoller, M. D. & Ruoff, R. S. Reduced graphene oxide/tin oxide composite as an enhanced anode material for lithium ion batteries prepared by homogenous coprecipitation. *J. Power Sources* **196**, 6473−6477 (2011).
12. Zhao, B. *et al.* Bivalent tin ion assisted reduction for preparing graphene/SnO_2 composite with good cyclic performance and lithium storage capacity. *Electrochim. Acta* **56**, 7340−7346 (2011).
13. Wang, X. *et al.* N-doped graphene-SnO_2 sandwich paper for high-performance lithium-ion batteries. *Adv. Funct. Mater.* **22**, 2682−2690 (2012).
14. Jiang, Y., Yuan, T., Sun, W. & Yan, M. Electrostatic spray deposition of porous SnO_2/graphene anode films and their enhanced Lithium-storage properties. *ACS Appl. Mater. Inter.* **4**, 6216−6220 (2012).
15. Yang, S., Yue, W., Zhu, J., Ren, Y. & Yang, X. Graphene-based mesoporous SnO_2 with enhanced electrochemical performance for lithium-ion batteries. *Adv. Funct. Mater.* **23**, 3570−3576 (2013).
16. Song, H., Li, N., Cui, H. & Wang, C. Enhanced capability and cyclability of SnO_2-graphene oxide hybrid anode by firmly anchored SnO_2 quantum dots. *J. Mater. Chem. A* **1**, 7558−7562 (2013).
17. Ye, F., Zhao, B., Ran, R. & Shao, Z. Facile mechanochemical synthesis of nano SnO_2/graphene composite from coarse metallic Sn and graphite oxide: an outstanding anode material for lithium-ion batteries. *Chem.-Eur. J.* **20**, 4055−4063 (2014).
18. Guo, Q. & Qin, X. Flower-like SnO_2 nanoparticles grown on graphene as anode materials for lithium-ion batteries. *J. Solid State Electrochem.* **18**, 1031−1039 (2014).
19. Etacheri, V. *et al.* Ordered network of interconnected SnO_2 nanoparticles for excellent lithium-ion storage. *Adv. Energy Mater.* **5**, 10.1002/aenm.201401289 (2015).
20. Birrozzi, A. *et al.* High-stability graphene nano sheets/SnO_2 composite anode for lithium ion batteries. *Electrochim. Acta* **137**, 228−234 (2014).
21. Dong, Y. *et al.* Dually fixed SnO_2 nanoparticles on graphene nanosheets by polyaniline coating for superior lithium storage. *ACS Appl. Mater. Inter.* **7**, 2444−2451 (2015).

22. Botas, C., Carriazo, D., Singh, G. & Rojo, T. Sn- and SnO_2-graphene flexible foams suitable as binder-free anodes for lithium ion batteries. *J. Mater. Chem. A* **3**, 13402−13410 (2015).
23. Tan, C., Zhao, S., Yang, G., Hu, S. & Qin, X. Facile and surfactant-free synthesis of SnO_2-graphene hybrids as high performance anode for lithium-ion batteries. *Ionics* **21**, 987−994 (2015).
24. Zhang, Z., Wang, L., Xiao, J., Xiao, F. & Wang, S. one-pot synthesis of three-dimensional graphene/carbon nanotube/SnO_2 hybrid architectures with enhanced lithium storage properties. *ACS Appl. Mater. Inter.* **7**, 17963−17968 (2015).
25. Zhu, Y., Guo, H., Zhai, H. & Cao, C. Microwave-assisted and gram-scale synthesis of ultrathin SnO_2 nanosheets with enhanced lithium storage properties. *ACS Appl. Mater. Inter.* **7**, 2745−2753 (2015).
26. Gong, C. *et al.* Green synthesis of 3D SnO_2/graphene aerogels and their application in lithium-ion batteries. *RSC Adv.* **5**, 39746−39751 (2015).
27. Li, W., Yoon, D., Hwang, J., Chang, W. & Kim, J. One-pot route to synthesize SnO_2-Reduced graphene oxide composites and their enhanced electrochemical performance as anodes in lithium-ion batteries. *J. Power Sources* **293**, 1024−1031 (2015).
